# Changes in Nutritional Status Impact Immune Cell Metabolism and Function

**DOI:** 10.3389/fimmu.2018.01055

**Published:** 2018-05-16

**Authors:** Yazan Alwarawrah, Kaitlin Kiernan, Nancie J. MacIver

**Affiliations:** ^1^Department of Pediatrics, Duke University Medical Center, Durham, NC, United States; ^2^Department of Immunology, Duke University Medical Center, Durham, NC, United States; ^3^Department of Pharmacology and Cancer Biology, Duke University Medical Center, Durham, NC, United States

**Keywords:** malnutrition, obesity, T cells, cellular metabolism, inflammation, leptin

## Abstract

Immune cell function and metabolism are closely linked. Many studies have now clearly demonstrated that alterations in cellular metabolism influence immune cell function and that, conversely, immune cell function determines the cellular metabolic state. Less well understood, however, are the effects of systemic metabolism or whole organism nutritional status on immune cell function and metabolism. Several studies have demonstrated that undernutrition is associated with immunosuppression, which leads to both increased susceptibility to infection and protection against several types of autoimmune disease, whereas overnutrition is associated with low-grade, chronic inflammation that increases the risk of metabolic and cardiovascular disease, promotes autoreactivity, and disrupts protective immunity. Here, we review the effects of nutritional status on immunity and highlight the effects of nutrition on circulating cytokines and immune cell populations in both human studies and mouse models. As T cells are critical members of the immune system, which direct overall immune response, we will focus this review on the influence of systemic nutritional status on T cell metabolism and function. Several cytokines and hormones have been identified which mediate the effects of nutrition on T cell metabolism and function through the expression and action of key regulatory signaling proteins. Understanding how T cells are sensitive to both inadequate and overabundant nutrients may enhance our ability to target immune cell metabolism and alter immunity in both malnutrition and obesity.

## Introduction

Nutritional imbalance is a major challenge for living organisms to achieve systemic homeostasis and maintain normal physiology. Mammals have developed processes to control systemic nutrient utilization and storage. For example, excess nutrients are converted and stored in adipose tissue, liver, and muscle during times when nutrients are abundant. By contrast, stored nutrients are metabolized to provide energy and building blocks to maintain vital physiological processes when nutrient availability is low.

From these processes, adipose tissue volume changes in response to under- or overnutrition. This change in adipose tissue volume, in turn, influences the secretion of hormones and cytokines from adipose tissue (adipocytokines). Many of these adipocytokines have important immune signaling functions which can influence immune cell biology and alter immune response.

Here, we will review the effects of nutritional changes on hormones and cytokines that influence immune cell function and metabolism in mouse models, and we will specifically examine how nutritionally regulated changes in immune cells alter immunity in the context of both autoimmune disease and infection response in both human and animal studies. We will also highlight some of the promising metabolic targets that may be useful in the development of novel treatments for immunity-related disorders.

## Key Signaling Molecules Altered in Response to Changes in Nutritional Status

Many cytokines and hormones are changed in response to over- or undernutrition. One of the earliest reports of this is with tumor necrosis factor-alpha (TNF-α). TNF-α can be secreted from adipose tissue, and its expression is increased during obesity but decreased following weight loss (1–3). TNF-α is a well-known pro-inflammatory cytokine that is essential for the acute phase reaction. Studies from the 1990s demonstrated that TNF-α was essential for the development of insulin resistance in high-fat diet-induced obesity in mice (1) and that deletion of TNF-α protected high-fat diet-induced obese mice from developing insulin resistance (4). TNF-α can be secreted from both adipose tissue-localized macrophages, which are increased in obesity, and from adipocytes (5, 6). Following these early studies showing the importance of TNF-α in mediating metabolic disease in obesity, multiple other cytokines and hormones were also found to play a similar role.

Interleukin-6 (IL-6) is another cytokine that is secreted by many immune and non-immune cells in the adipose tissue including adipocytes, macrophages, pre-adipocytes, and T cells, in response to tissue damage (7). IL-6 has broad pleiotropic functions leading to the expansion of many types of immune cells including B cells and T cells (8, 9). IL-6 can signal through binding to the IL-6 receptor dimerized with gp130 on the surface of cells; however, IL-6 effects can also be mediated through trans-signaling, in which IL-6 binds to soluble IL-6 receptor, thereby permitting IL-6 to act on any cell that expresses gp130. In that way, IL-6 receptor trans-signaling contributes to the broad pleiotropic effect of IL-6 (10). In general, IL-6 promotes T cell survival and resistance to apoptosis (11). IL-6 also has a pro-inflammatory role promoting CD4+ T cell differentiation to the Th17 or Th1 lineages, which produce the pro-inflammatory cytokines IL-17 and interferon gamma (IFN-γ), respectively (12, 13). Increased IL-6 levels have been reported in obesity in both humans and rodents (14, 15). Pan-blocking of IL-6 signaling using an anti-IL-6 antibody (MR16-1) has been shown to ameliorate insulin resistance and reduce liver-fat accumulation in high-fat diet-fed mice (16). Blocking IL-6 trans-signaling by using gp130Fc soluble protein was found to block adipose tissue macrophage recruitment in high-fat diet-fed mice, but did not inhibit insulin resistance (17). Recently, it was reported that selective blocking of IL-6 signaling in T cells improves glucose homeostasis and ameliorates liver steatosis in high-fat diet-fed mice, but only early in the development of obesity (18).

Leptin is another well-described adipocytokine known to influence immune cells. Leptin is a hormone secreted by adipocytes in proportion to adipocyte mass and is therefore increased in obesity and decreased in malnutrition. Leptin is best known for its role in influencing systemic metabolism by signaling in the hypothalamus to suppress appetite and increase energy expenditure (19). However, leptin can also communicate energy status to other systems in the body, including the immune system (20). In that role, leptin was found to have an important developmental function in the maturation of hematopoietic cells on which the leptin receptor (LepR) is expressed (21). In addition to this developmental function, leptin deficiency has also been associated with the loss of cell-mediated immunity (22). The first reported immune function for leptin was its ability to regulate macrophage phagocytosis and pro-inflammatory cytokine production: lack of leptin or its receptor was found to reduce phagocytic activity and the production of both IL-6 and TNF-α (23). In addition, LepR is expressed on the surface of T cells (24), and deletion of LepR on T cells leads to a marked decrease in T cell number and function as well as polarization to Th1 and Th17 cell subsets, ultimately leading to immune deficiency characterized by an increased susceptibility to intracellular infections (25–27).

In addition to TNF-α, IL-6, and leptin, many other hormones and cytokines are influenced by changes in systemic metabolism and are summarized in Table 1.

**Table 1:** Key immune signaling molecules that change in response to nutritional status.

Molecule	Class	Obesity	Malnutrition	Secreted by	Immune function	Reference
Leptin	Adipocytokine	Increased	Decreased	Adipocytes	Pleiotropic hormone with many targets and many functions in immune cells Induces Th1 and Th17 polarization	(28)

Adiponectin	Adipocytokine	Decreased	Conflicting reports—may be context-dependent	Adipocytes	Polarization of monocytes and macrophages toward M2 phenotype Suppresses NK cell, eosinophil, neutrophil, γδ T cell, and dendritic cell activation and inflammatory cytokine production	(29)

Resistin	Adipocytokine	Increased	No clear correlation; more investigation needed	Adipocytes, macrophages	Stimulates the production of TNF-α and IL-12 in macrophages	(30)

Visfatin	Adipocytokine	Increased	No clear correlation; more investigation needed	Adipocytes	Stimulates the production of TNF-α, IL-1β, and IL-6	(30, 31)

TNF-α	Cytokine	Increased	Mixed results, may depend on context	Adipocytes	Neutrophil chemotaxis	(4, 15, 32–34)

IL-1β	Cytokine	Increased	Mixed results, may depend on context	Non-adipocyte cells in adipose tissue	Stimulates macrophage activity	(35, 36)

IL-6	Cytokine	Increased	Decreased	Adipocytes, macrophages	Recruitment of macrophages; polarization toward pro-inflammatory classically activated macrophages	(15, 37)

IL-8	Cytokine	Increased	Decreased	Adipocytes, macrophages	Induces neutrophil chemotaxis	(38–40)

IL-10	Cytokine	Mixed—may be context-dependent	Increased	Treg cells, iNKT cells, DCs, adipocytes, macrophages	Broad anti-inflammatory function	(41–43)

IL-33	Cytokine	Decreased	Increased	DCs, macrophages, epithelial cells	Maintains adipose tissue-resident Treg cell function, promotes Th2 response, promotes alternatively activated macrophage polarization	(44, 45)

IL-1RA	Cytokine	Increased	Unknown	Macrophages, epithelial cells	Inhibits IL-1α and IL-1β activity	(46–48)

MCP-1	Chemokine	Increased	Increased	Macrophages, adipocytes	Macrophage recruitment	(39, 49)

MIF	Chemokine	Increased	Decreased	Adipocytes, lymphocytes	Inhibits macrophage migration	(50)

MIP-1α	Chemokine	Increased	Unknown	Adipocytes	Enhances macrophage migration	(51)

MIP-1β	Chemokine	Increased	Unknown	Adipocytes	Enhances macrophage migration	(51)

## Immune Cells Affected by Changes in Nutritional Status

The hormone and cytokine changes seen in response to obesity and malnutrition are closely linked to changes in immune cell populations. Several types of immune cells residing in the adipose tissue are affected by changes in the above-listed cytokine and hormone levels and in turn contribute to altered cytokine production in states of under- or overnutrition. These adipose tissue-localized immune cells can be affected by changes in nutritional status through both paracrine effects (due to their proximity to adipocytes) and systemic/endocrine effects of secreted adipose factors.

Macrophages comprise more than 50% of adipose tissue-resident immune cells and are, therefore, the most abundant immune cells in the adipose tissue (52). During obesity, an influx of macrophages into the adipose tissue takes place in response to the secretion of monocyte chemoattractant protein-1 from the adipose tissue (53). These macrophages become polarized into pro-inflammatory, classically activated macrophages (previously termed M1 macrophages) in response to IFN-γ, which is secreted by effector T cells (Teff cells) and other immune cells in the adipose tissue (54). This increase in inflammatory macrophage population leads to an increase in TNF-α secretion in addition to other inflammatory molecules secreted by inflammatory macrophages, including IL-1β, IL-6, and IL-12. Adipose tissue macrophages in obesity contribute to the formation of the crown-like structure that forms around necrotic adipocytes, a very distinctive histological feature of the adipose tissue during obesity. In lean individuals, alternatively activated macrophages (previously termed M2 macrophages) secrete anti-inflammatory cytokines including IL-10, IL-4, and IL-1 receptor agonists which promote immune modulatory functions (55). The shift in macrophage populations during obesity plays a central role in the maintenance of inflammation and the rise of obesity-associated pathologies including insulin resistance and non-alcoholic fatty liver disease.

In addition to macrophages, other smaller populations of innate immune cells are found in the adipose tissue and change in number and function in response to obesity. Neutrophils and mast cells are found to increase and become activated in the adipose tissue during obesity (56). By contrast, eosinophil numbers decrease in adipose tissue during obesity (57), which is relevant because eosinophils secrete IL-4, a cytokine that helps maintain the alternatively activated population of macrophages within the adipose tissue (57).

Several lymphocyte populations are also found in the adipose tissue. B lymphocytes (B cells) have been found to accumulate in the adipose tissue during obesity (58). Although B cells are best known for the production of antibodies, they also express inflammatory cytokines, such as IL-2 and IL-12, which influence T cell differentiation into Th1 versus Th2 cells (59). Adipose tissue also harbors a large population of natural killer T (NKT) cells (60); these cells are known for the expression of an invariant form of the T cell receptor (TCR) that interacts with a lipid antigen presenting protein, CD1d, which is highly expressed on adipocytes (61). NKT cells secrete different types of cytokines depending on the lipid antigen presented by CD1d. During normal weight conditions, NKT cells modulate inflammation by secreting anti-inflammatory cytokines such as IL-4 and IL-10 (62). In obesity, NKT cells decrease in number, at the same time adipocytes express lower levels of CD1d. This change altogether reduces the amount of anti-inflammatory cytokines secreted by NKT cells and contributes to the complications of obesity (62).

T lymphocytes (T cells) represent the most abundant lymphocyte population and second most abundant immune cell in the adipose tissue behind macrophages (63). Both CD4+ and CD8+ T cells are found in the adipose tissue. During obesity, the proportion of adipose CD8+ T cells to CD4+ T cells increases (64). In addition, proportions of inflammatory CD4+ T cell subsets increase in obesity, whereas regulatory T cells (Treg cells) decrease (64, 65). This change in T cell populations during obesity contributes to the pro-inflammatory state of the adipose tissue: both CD8+ T cells and pro-inflammatory CD4+ Th1 cells express the pro-inflammatory cytokine IFN-γ, whereas effector CD4+ Th17 cells express the pro-inflammatory cytokine IL-17.

During normal physiological conditions, the adipose tissue represents a major depot of Treg cells in the body (66, 67). These cells represent more than 50% of CD4+ T cells in lean adipose tissue (66). Treg cells are responsible for suppressing inflammation through the secretion of anti-inflammatory cytokines such as TGF-β and IL-10 (58, 63). During obesity, the proportion of adipose tissue Treg cells decreases dramatically as adipose tissue volume increases. Due to their role in maintaining self-tolerance and in dampening excessive inflammatory response, the reduction in Treg cell number during obesity (68) induces a significant shift in immune cell populations and cytokine production toward a pro-inflammatory state (65). Paradoxically, adipose tissue Treg cells were also found to contribute to age-associated insulin resistance, and deletion of Treg cells could protect against age-associated, but not obesity-associated, insulin resistance (69).

In contrast to the effects of obesity on immune cells, malnutrition leads to a *decrease* in immune cell number. This has been shown particularly in the case of T cells: mice fasted 48 h had large and significantly decreased thymocyte and splenocyte counts compared to fed control mice (26, 70, 71). Both total T cell and CD4+ T cell numbers from spleens of fasted mice were decreased by 40–50% compared to fed control animals (26, 71). Other studies have shown that mice fed a protein-deficient diet had atrophic spleens and decreased T cell numbers compared to chow-fed control mice (72, 73). A similar finding was seen in human studies. Malnourished children had decreased CD4+ and CD8+ T cell numbers in whole blood compared to well-nourished children (74). Moreover, childhood malnutrition causes atrophy of primary lymphoid organs, leading to reduced T and B cell numbers and a generalized state of leukopenia (75). These reductions in immune cell number in malnutrition contribute to functional deficiencies, which will be discussed in further detail below.

## Effect of Nutritional Status on Immune Cell Metabolism

Although it is clear that systemic metabolism influences immune cell function, we are only just starting to understand how changes in nutrition can influence metabolism at the cellular level. This is an important consideration, as immune cell metabolism and immune cell function are intrinsically tied. Previous studies have demonstrated a link between cellular metabolism and function for several types of immune cells (76, 77), but we will focus our discussion here on T cells. Multiple studies have now shown that changes in T cell metabolism can influence T cell differentiation and function, whereas changes in T cell function can likewise influence T cell metabolism.

The energy requirement of naïve T cells performing immune surveillance is satisfied through oxidative phosphorylation of lipids, amino acids, and glucose-derived pyruvate to ATP in the mitochondria (78). This process is highly efficient at producing ATP, but does not provide biosynthetic precursors that are necessary for proliferation or growth. Naïve T cells are arrested in the G_0_ stage of the cell cycle and this state of homeostatic quiescence is actively maintained (79). Without TCR stimulation, CD4+ T cells fail to undergo homeostatic proliferation, downregulate Glut1, and die from apoptosis (80, 81). Following activation, however, T cells need to rapidly grow, proliferate, and generate cytokines to direct a functional immune response. Given the growth and proliferation requirement of an activated T cell, these cells must be prepared to increase the biosynthesis of cellular products including lipids, proteins, and nucleotides which are needed for rapid cell division (78), and for these reasons, a metabolic switch is required.

Upon activation, the metabolic state of T cells resembles that of cancer cells (82). These rapidly proliferating cells increase glucose uptake, glycolysis, and reduction of pyruvate to lactate even in the presence of oxygen, a process aptly named aerobic glycolysis (83). Warburg noticed this effect in his early studies of blood leukocytes, and more recent studies have confirmed the “Warburg effect” in thymocytes and T cells (84, 85). A state of rapid ATP usage and massive biosynthetic requirement make the process of glycolysis a more efficient way for cancer cells and activated T cells to proliferate. TCA cycle intermediates can be used as precursors in biosynthetic pathways to support the growing need for lipid, protein, and nucleotide synthesis that precedes cellular division (78). Conversion of pyruvate to lactate ensures that reducing equivalents of NAD+ are restored, allowing the process of glycolysis to continue (83).

The upregulation of the glycolytic metabolic program in activated T cells is controlled by several key signaling pathways and transcription factors. Both TCR signaling and signaling through CD28 induce the activation of the phosphatidylinositol-4,5-bisphosphate 3-kinase (PI3K)/Akt pathway, which is partially responsible for the upregulation of a glycolytic metabolic program (80, 86). The PI3K/Akt pathway leads to the activation of mammalian target of rapamycin (mTOR) which forms two functional complexes: mammalian target of rapamycin complex 1 (mTORC1) and mammalian target of rapamycin complex 2 (mTORC2). mTOR is an important integrator of environmental signals, allowing the cell to respond to signals from the TCR, co-stimulation, cytokines, and nutrient availability (87–89). The activation of Akt and mTOR has been shown to lead to an increase in aerobic glycolysis by increasing the transcription of glycolytic genes and transcription factors including c-Myc and hypoxia-inducible factor (HIF)-1α (87, 88). In addition, constitutive Akt expression leads to an increase in surface expression of the glucose transporter Glut1 and thereby increases glucose uptake, whereas blocking Akt or PI3K decreases T cell glucose uptake. The inhibition of mTORC1 by treatment with rapamycin has been shown to prevent T cell growth and proliferation (90, 91). Knockout of the mTOR inhibitor AMPKα1 decreases the ability of T cells to respond to metabolic stress and decreases their ability to transition between anabolic and catabolic metabolisms (92). mTOR also regulates protein translation as part of mTORC1 through interaction with the translation initiation factor 4E-binding protein (4E-BP) and p70s6K. By phosphorylating 4E-BP and p70s6K, mTOR activates these two proteins to increase protein translation in the cell (88).

At the transcriptional level, c-Myc is responsible for regulating glucose and glutamine metabolism. Specifically, c-Myc has been shown to upregulate multiple glycolytic genes upon T cell activation, including Glut1, hexokinase2 (HK2), and pyruvate kinase muscle isozyme 2 (93). Glut1 is a critical glucose transporter expressed in T cells and is upregulated upon activation. Glut1 can be stored in vesicles intracellularly and transported to the cell surface to increase glucose uptake following T cell activation, and Glut1 transcription and translation are increased following T cell activation (94). In cancer cells, c-Myc has been shown to control the transition to glycolytic metabolism in hypoxic conditions along with HIF-1-α (95). c-Myc is upregulated following T cell activation and has been demonstrated by several groups to induce cell cycle progression and glycolytic metabolism (96, 97). Without this transition, T cells would not be able to exit G_0_ and enter the rapid expansion phase of the immune response. This has been confirmed in a T cell specific knockout of c-Myc, in which T cells fail to proliferate and were unable to upregulate glycolytic metabolism (95). c-Myc also upregulates glutaminolysis which can feed into anaplerosis, so that TCA cycle intermediates can be used for biosynthesis. For example, citrate is a TCA cycle intermediate that can be used as a precursor for lipid synthesis (95). HIF-1-α is also increased upon T cell activation and has been shown to increase the expression of glycolytic genes along with c-Myc (95, 98). HIF-1-α is also important in the differentiation of T cells, particularly toward Th17 cells.

Differentiation of CD4+ T cells into functionally distinct subsets is associated with alterations in the cellular metabolic phenotype. In general, Teff cells are pro-glycolytic and depend heavily upon an increased glucose uptake, as well as glutamine metabolism, to fuel effector function, as described above. Inhibition of glycolysis prevents differentiation into these pro-inflammatory subsets (99). Regulatory T cells (Treg) and memory T cells (Tmem), however, have a decreased glucose metabolism and predominantly utilize lipid oxidation to fuel suppressive and memory function, respectively (100–102). Although Treg cells have a decreased glucose metabolism in comparison to Teff cells, glycolysis is essential for Treg cell migration into sites of inflammation (103).

The first evidence of T cell differentiation depending on metabolism came from studies using the drug rapamycin. Treatment with rapamycin prevented T cell growth and proliferation and promoted differentiation of Treg cells, rather than Teff cells (90). Further evidence came from studies using whole-body or T cell-specific knockout of mTOR, in which only Treg cells were produced under activation conditions (104). Failure to produce Teff cells in the absence of mTORC1 demonstrates that glycolytic metabolism, as driven by mTORC1, is required for Teff cell differentiation and cytokine production. Moreover, increasing the activity of AMPK by treatment with metformin increased Treg cell numbers and decreased Teff cells, further demonstrating that blocking glycolytic metabolism promotes the Treg cell lineage (100). Metformin-treated T cells also had lower Glut1 levels upon activation, demonstrating a decreased glycolytic state compared to untreated T cells in these studies (100).

Not only does the activity of mTORC1 promote Teff cell differentiation over Treg cells but differential activity of mTORC1 and mTORC2 promotes distinct Teff subsets (78, 98, 105). mTORC1 activity is upregulated in Th1 and Th17 cells, whereas Th2 cells show an increased activity of mTORC2. Treg cells also demonstrate an increased AMPK activity, which leads to inhibition of mTOR. Tmem express both TRAF6 and AMPK, which promote lipid oxidation and thereby suppress the glycolytic phenotype of activated Teff cells (102).

### Leptin Promotes Glycolytic Metabolism in Activated T Cells

One well-established connection between systemic nutritional status and immune cell metabolism is through leptin. As mentioned above, leptin is secreted by adipocytes in proportion to adipocyte mass and leptin levels, thereby trending with nutritional status (106). In states of malnutrition or following fasting, circulating leptin levels are decreased, whereas in obesity, leptin levels are increased. Leptin acts directly on CD4+ T cells through the LepR to direct changes in T cell metabolism and function (26, 27, 107–110). Since T cell metabolism and function are intimately linked, any change in immune cell metabolism can lead to a change in the function of that cell, altering cellular proliferation, differentiation, and cytokine production. Leptin has been shown to promote both CD4+ T cell inflammatory cytokine production and glucose metabolism. Indeed, T cells unable to respond to leptin had impaired upregulation of glucose uptake and glycolysis following T cell activation (26). In the context of malnutrition, fasting-induced hypoleptinemia caused activated CD4+ T cells to produce less inflammatory cytokines IFN-γ and IL-2 (26, 27). That functional defect did not extend to naïve T cells or Treg cells, however, presumably because those cells do not depend on increased glycolytic metabolism to fuel immune surveillance or regulatory function. In subsequent studies, Th17 cells from fasted mice were found to be functionally deficient and metabolically less glycolytic, whereas Treg cells from fasted mice did not experience a functional or a metabolic defect (27). The metabolic status of Th17 cells derived from fasted mice was assessed by extracellular flux analysis, and these cells were found to have a decreased lactate production as well as a decreased mitochondrial respiration compared to Th17 cells from *ad libitum* fed mice. The functional and metabolic defects of Th17 cells were restored when fasted mice received leptin injections or when T cells isolated from fasted mice were activated in the presence of leptin *in vitro*. Leptin can affect many types of immune system; however, these effects of leptin on T cells were shown to be cell-intrinsic, as Th17 cells from T cell-specific LepR conditional knockout mice showed a decreased glucose uptake and glycolysis as well as a decreased glycolytic enzyme expression, whereas glucose metabolism and function of Treg cells from T cell-specific LepR conditional knockout mice were unaffected. Altogether, these studies show that leptin is a systemic hormone that communicates nutritional status to immune cells by directly increasing Teff cell glucose metabolism and thereby fueling Teff cell function.

The effect of leptin on immune cells is now well documented; however, other nutritionally regulated hormones may also mediate the communication between systemic nutritional status and immune cell function. One such candidate hormone is insulin. Insulin is secreted from pancreatic beta cells following an increase in blood glucose; however, insulin levels also become elevated in states of obesity due to insulin resistance of metabolic tissues including muscle and adipose tissue. Insulin is best known for its role in promoting glucose uptake and glycolysis in metabolic tissues through its ability to upregulate surface expression of the glucose transporter Glut4 and increase the activities of the glycolytic enzymes hexokinase and phosphofructokinase (111–116). Insulin is also able to regulate lipid and protein metabolism. Interestingly, insulin receptors are expressed on activated CD4+ T cells (117). One recent study in rats showed that inducible knockdown of insulin receptor in T cells led to a decreased T cell glucose metabolism and cytokine production (118). Further studies are needed to define the mechanism by which changes in systemic nutrition alter circulating levels of insulin and thereby influence immune cell metabolic and functional processes.

## Effect of Nutritional Status on Immune-Mediated Disease

### Autoimmunity

Given the role of leptin in regulating the balance between Teff and Treg cells, multiple studies have examined the effect of leptin on autoimmune diseases. One well-studied example is the autoimmune disease multiple sclerosis (MS). The autoimmunity of MS depends upon the activation of Teff, particularly Th1 and Th17 cells, which produce inflammatory cytokines that promote inflammation. Conversely, Treg cells influence immune response by suppressing inflammation caused by Teff and other inflammatory immune cells and thereby protect against autoimmunity. The balance of Teff (Th1, Th17) to Treg cells in MS is, therefore, a critical determinant of inflammation and autoimmune disease. Multiple human studies have shown that active MS is associated with a decreased Treg cell number and proliferation (119–121).

Nutritional status appears to influence MS susceptibility. Clinical studies have shown an association between adolescent obesity and an increased risk of developing MS, particularly in women (122–125). Consistent with that observation, leptin levels have been found to be increased in patients with MS in both serum and cerebrospinal fluid and are associated with increased inflammatory cytokines (119, 120, 126). In addition, LepR expression was increased on the surface of CD8+ T cells from relapsing–remitting MS patients in relapse, as compared to patients in remission or controls (127).

The role of leptin in promoting T cell inflammation has also been well described in a mouse model of MS, experimental autoimmune encephalomyelitis (EAE). Serum leptin levels have been shown to be increased in EAE and correlate with disease severity (128, 129). Early studies in EAE showed that leptin injections worsened EAE disease in female mice and increased disease susceptibility in male mice, while promoting inflammatory cytokine release (130). Moreover, leptin-deficient mice were found to be resistant to EAE, but this protection was lost when mice were treated with recombinant leptin protein (128, 131). Leptin-neutralizing antibodies likewise protected against T cell response and EAE disease in mice (107).

Since leptin levels decrease with fasting and calorie restriction, the effect of fasting on EAE disease severity has been examined by several groups. Fasting-induced hypoleptinemia resulted in a reduced EAE disease severity (27, 129, 132, 133). This fasting-induced protection against EAE was reversed, in part, when fasted mice received leptin injections (27). Moreover, T cells recovered from draining lymph nodes in fasted EAE mice showed a decreased production of the Th1 and Th17 cytokines IFN-γ and IL-17, respectively, as well as a decrease in the expression of glycolytic proteins Glut1 and HK2 (27); again, these fasting-induced changes in Teff cell cytokine production and glucose metabolism were reversed in T cells from fasted mice receiving leptin injections.

Leptin signaling has also been implicated in several other autoimmune diseases. In systemic lupus erythematosus (SLE), leptin levels have been reported to be elevated in human patients and correlate with severity in a mouse model of the disease (134, 135). Decreasing leptin signaling through genetic knockout or antibody blockade protected against disease and increased Treg cell numbers in SLE mice (134). Additional studies in SLE models demonstrated an increased Th17 response, which could be attenuated with the neutralization of leptin (110). Leptin has also been reported to increase Th17 activity in Hashimoto’s thyroiditis and collagen-induced arthritis (108, 109). This provides further evidence for the role of leptin in promoting Th17 differentiation and activation, thus promoting autoimmune pathology. Although not strictly an autoimmune disease, it is also notable that in an allogeneic skin-transplant model, leptin-deficient mice showed an increase in graft survival relative to wild-type mice (136). Altogether, leptin signaling appears to provide an important link between nutritional status and autoimmune disease through its effects on T cell metabolism and function.

### Protective Immunity

Like any other physiological system, the development of the immune system is affected by nutritional status. One salient example for this is the thymic atrophy and increase in thymocyte apoptosis observed during malnutrition early in life (137–139). This has a devastating effect on the ability of the immune system to mount a successful immune response to infection. Moreover, many epidemiological studies have shown dysfunction in both innate and adaptive immunity during malnutrition (138). This explains the increased susceptibility to many kinds of infection in the malnourished individuals, such as influenza, tuberculosis, *Streptococcus pneumonia* and gastrointestinal infections (140–143), and the poor response to vaccines (138).

Obesity, however, is also associated with susceptibility to a number of infections (144). One example of this is with influenza. This was first reported when studies on the H1N1 strain of influenza showed a connection between obesity and poor disease outcome (145). Indeed, obesity was found to be a risk factor for developing H1N1 infection and was associated with a longer length of stay in the intensive care unit and with higher rates of mortality (146–148). Following the discovery that obesity increased risk and mortality from H1N1 flu, obesity that was subsequently found to be is an independent risk factor for increased morbidity and mortality from *all* strains of influenza.

In addition to increased susceptibility to influenza, individuals with obesity are also at an increased risk from other infections: obese individuals have an increased risk of developing complications such as sepsis, pneumonia, and bacteremia following surgical procedures (149); they are more prone to *Helicobacter pylori* infection (150), and obese children were found to have three times greater risk of being asymptomatic carriers of *Neisseria meningitides* (151). In addition, obesity is associated with a lower antibody response to select vaccinations including influenza, hepatitis B, and tetanus (152).

In the case of malnutrition, the lack of protective immunity can be easily traced back to the developmental defects associated with inadequate nutrients and the lack of nutritional signals such as leptin that are critical for fueling immune cell proliferation and function. On the other hand, the susceptibility to infection and poor vaccine response associated with obesity seems unexpected when factoring in that obesity is accompanied with a low-grade inflammation and constant activation of immune cells. In the case of influenza, obesity has been found to be associated with impaired memory response (153). One possible explanation for this is that the systemic metabolic environment in obesity promotes a cellular metabolism in immune cells which supports short-lived effector cells over the generation of long-term memory cells.

## Targeting Immune Metabolism for Disease Treatment

As we have documented here, many signaling cascades are affected by nutritional status and subsequently have an effect on immune cell metabolism and function. For that reason, immune cell metabolism represents an attractive target to improve response in both malnutrition and obesity. Here, we will highlight several signaling molecules and metabolic enzymes that play central roles in the metabolic reprogramming of immune cells and are critical for mounting an immune response and initiating inflammatory reaction. These metabolic molecules serve as potential targets to reverse the effects of obesity and malnutrition on immunodeficiency and inflammation, respectively.

### Glut1

Activated immune cells are dependent on a glycolytic metabolism to fuel rapid ATP production and provide biosynthetic materials for growth and proliferation. For that reason, activated immune cells require a large influx of glucose to fuel glycolysis (154). This suggests the glycolysis pathway as a potential target for the control of inflammation. Multiple reports have shown that the inhibition of glycolysis by 2-deoxyglucose (2-DG) can block CD4+ T cell proliferation, inflammatory macrophage polarization, and B cell survival (155–157). Although 2-DG shows a potent anti-inflammatory activity and has shown tolerability in clinical trials for the treatment of prostate cancer, cardiac adverse reactions to 2-DG were reported (158, 159), and alternative targets for the inhibition of glucose metabolism are required.

Glucose transport represents the most upstream, rate-limiting step for glycolysis. There are approximately 13 members of the glucose transporter family expressed to various extents in different tissues. The best-described glucose transporter is Glut4, which is an insulin-sensitive glucose transporter expressed on metabolic tissues including muscle, adipose tissue, and liver. However, Glut4 is not expressed on T cells (160). Rather, T cell glucose uptake is largely dependent on the ubiquitously expressed glucose transporter Glut1. Glut1 expression is upregulated in classically activated macrophages, activated Teff cells, and B cells, all of which depend on Glut1 for an increased glucose uptake during activation (154, 160, 161). Glut1 may, therefore, provide a more appropriate target for blocking glycolysis in activated immune cells than 2-DG. Treating macrophages with the Glut1 inhibitor Fasentin was found to inhibit the production of IL-1β (162). Currently, there are several trials to synthesize more selective small molecule inhibitors for Glut1 (163–165). The bioavailable Glut1 inhibitor BAY-876 represents one of the most potent Glut1 inhibitors (IC50 = 2nM) and shows more than 100-fold selectivity against the other glucose transporters (164). Unpublished data from our laboratory showed the ability of BAY-876 to selectively inhibit glucose uptake in activated CD4+ T cells. Although there is limited information about targeting Glut1 in immune cells, the new generation of selective Glut1 inhibitors may have the potential to be used as a therapy for the control of inflammation. Caution must be taken with this class of inhibitors, though, as Glut1 is critical for glucose transport to the brain and Glut1 mutations have been described in disorders of seizures and developmental delay (166, 167).

### PFKFB3

One mechanism by which glycolysis is regulated is through the metabolite fructose-2,6-bisphosphate (F26BP), which is an allosteric activator of 6-phosphofructo-1-kinase: a rate-limiting enzyme in the glycolysis pathway that phosphorylates fructose 6-phosphate (F6P) to yield fructose 1,6-bisphosphate (168). The intracellular levels of F26BP are regulated by a family of bifunctional enzymes known as 6-phosphofructo-2-kinase/fructose-2,6-biphosphatase enzymes (PFKFB 1 through 4) which can convert F6P into F26BP and *vice versa* based on the phosphorylation levels of the enzyme (169). PFKFB3 expression is upregulated in activated macrophages and T cells (170, 171). In T cells, treatment with the PFKFB inhibitor 3-(3-pyridinyl)-1-(4-pyridinyl)-2-propen-1-one (3PO) has been shown to block both glycolysis and activation (171). *In vivo*, 3PO treatment of BALB/c mice challenged with methylated BSA was found to inhibit delayed-type hypersensitivity, highlighting the viability of targeting PFKFB activity for the control of inflammation (171). Unfortunately, the number of selective PFKFB small molecule inhibitors is limited; the only other PFKFB inhibitor, in addition to 3PO, is PFK15. PFK15 is a more potent PFKFB inhibitor than 3PO and showed antiproliferative activity in Jurkat T cell leukemia cells and several solid tumor-derived cell lines, but its effect on primary immune cells has yet to be investigated (172). Similar to targeting Glut1, targeting PFKFB activity may provide an additional tool to block glycolysis; however, more potent and selective inhibitors are needed, as are additional studies to investigate the effect of targeting such enzymes on immune function.

### BCAT1

Most activated immune cells are dependent on glycolysis for the rapid generation of energy, which limits the flow of pyruvate to the TCA cycle. However, TCA cycle intermediates are essential for proliferation and immune cell functions, and activated immune cells generally overcome this by metabolizing amino acids. This requires the upregulation of many genes involved in amino acid transport and catabolism.

Branched-chain aminotransferase (BCAT) is the enzyme responsible for the transamination of branched-chain amino acids (BCAAs): leucine, isoleucine, and valine. There are two isoforms of the BCAT enzymes: cytosolic BCAT1 and mitochondrial BCAT2. Transamination of BCAAs is the initial reaction toward the formation of branched-chain α-keto acids, which are decarboxylated to produce coenzyme A (CoA) derivatives (173). Leucine transamination leads to the formation of glutamate and α-ketoisocaproate. The α-ketoisocaproate molecule is subsequently metabolized to form acetoacetate and acetyl-CoA, which is then oxidized in the TCA cycle, similar to glutamate which enters the TCA cycle in the form of α-ketoglutarate.

Branched-chain aminotransferase 2 is highly expressed in many tissues including the kidneys, skeletal muscle, and tissues of the digestive system, while BCAT1 is expressed in a small number of tissues including placenta, adult brain, peripheral neurons, and a limited number of embryonic tissues (174). BCAT1 was found to be highly expressed in human monocyte-derived macrophages compared to BCAT2 (175). Blocking the activity of BCAT1 by the leucine analog ERG240 was found to inhibit the induction of cis-aconitate decarboxylase (IRG1) expression by LPS which is a crucial step for the activation for macrophages (175). ERG240 treatment was able to reduce oxygen consumption and glycolysis in human macrophages. Consistent with this phenotype, the administration of ERG240 ameliorated the severity of crescentic glomerulonephritis in rats and collagen-induced arthritis in mice (175).

### F1F0-ATPase

In contrast to acutely activated T cells being dependent on glycolysis, chronically activated T cells involved in autoimmune disease are more dependent on oxidative phosphorylation for energy production (176–178). This suggests that components of the oxidative phosphorylation pathway may be targets to block the expansion of autoreactive T cells in autoimmune diseases. The ATP synthase F1F0-ATPase catalyzes the final step of energy production of the respiratory chain in the mitochondria. F1F0-ATPase was found to be the target for the 1,4-benzodiazepine (Bz-423) which is reported to induce apoptosis of pathogenic lymphoid cells in lupus mouse models (179–181). Bz-423 was also reported to induce apoptosis of alloreactive T cells in a graft-versus-host disease model (182). Based on this, F1F0-ATPase gained special attention as a viable drug target for the treatment of autoimmune diseases. Currently, there are ongoing clinical trials for the treatment of inflammatory bowel disease and ulcerative colitis using the F1F0-ATPase small molecule inhibitor LYC-30937.

## Conclusion

**Figure 1:**
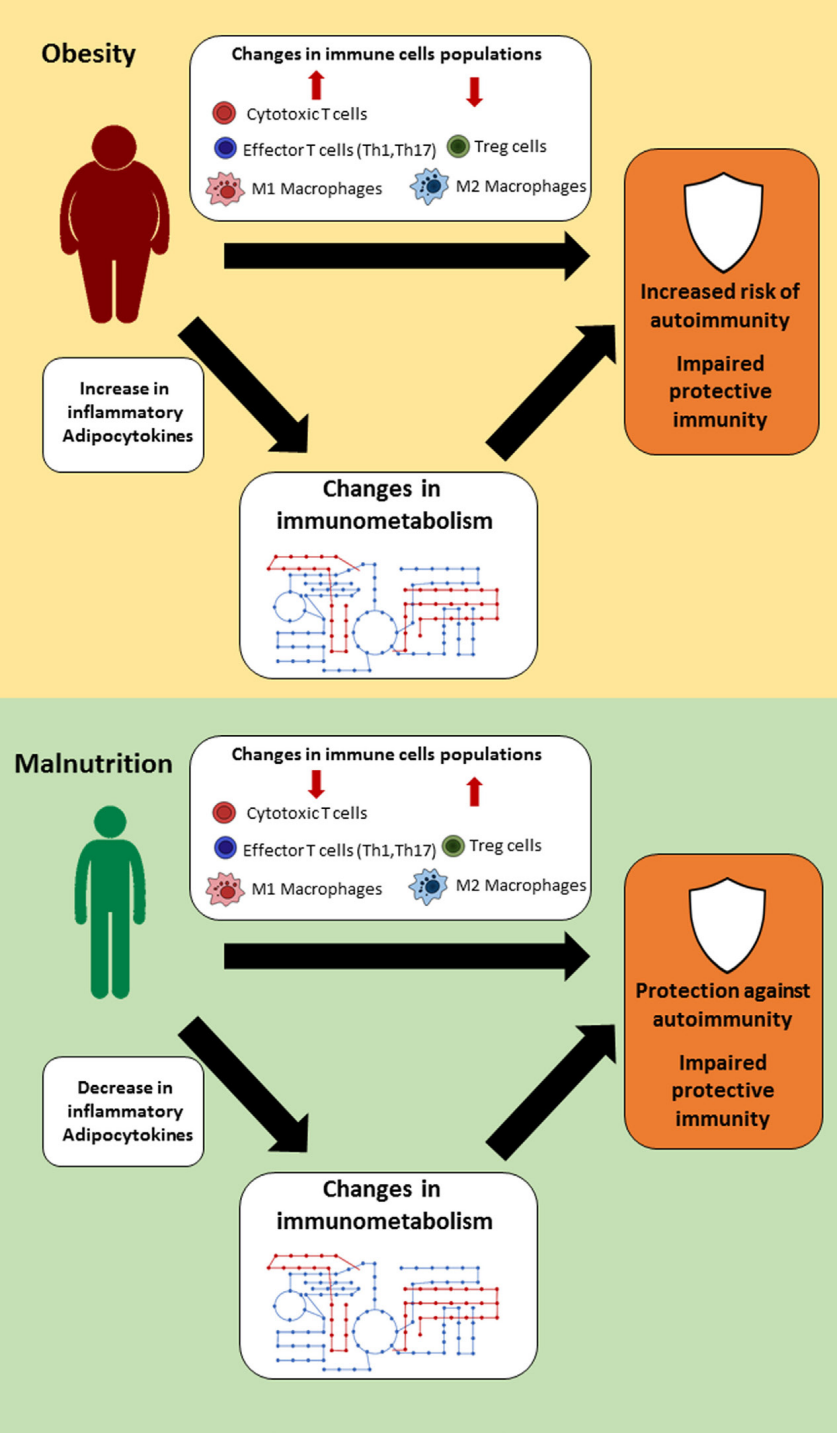
Suggested links between nutritional status, immune metabolism, and immune function. In settings of extreme nutritional status (obesity or malnutrition), changes in immune cell populations, hormones, and cytokine levels lead to alternations in immune cell metabolism, which thereby influence immune function.

Changes in nutritional status have a wide range of effects on the body, which can influence organ size, hormone, and cytokine levels, and immune cell populations and function. This link between nutrition and immunity is mediated, in part, by a select group of adipocytokines, such as leptin, which can influence immune cell number and function through its effects on cellular metabolism (Figure 1). For that reason, leptin has been identified as a key regulator of both protective immunity and autoimmunity in the context of nutritional disorders. Other cytokines and hormones likely play a similar key role in linking nutrition and immunity. In understanding the mechanisms by which nutrition influences immunity, we can identify targets to improve or normalize immunity in cases of under- or overnutrition. Currently, there are several small molecules under investigation that show promising preclinical results and the potential for restoring immune response in both malnutrition and obesity. In summary, more studies are needed to clarify the link between nutritional status, immune metabolism, and immune function; such knowledge may pave the way for the development of novel classes of therapies that can reverse the detrimental effects of the extremes of nutritional status on immunity.

## Author Contributions

All authors participated in the writing of this manuscript.

## Conflict of Interest Statement

The authors declare that the research was conducted in the absence of any commercial or financial relationships that could be construed as a potential conflict of interest.
